# Incident Arrhythmias Detected Using Implantable Loop Recorders in Obstructive Sleep Apnoea

**DOI:** 10.31083/RCM31308

**Published:** 2025-07-11

**Authors:** Hejie He, Ven Gee Lim, Nicholas Weight, Thomas Lachlan, Faizel Osman

**Affiliations:** ^1^Institute for Cardiometabolic Medicine (ICMM), University Hospital Coventry, CV2 2DX Coventry, UK; ^2^Warwick Medical School, University of Warwick, CV4 7HL Coventry, UK; ^3^Centre for Healthcare & Communities, Coventry University, CV1 5F Coventry, UK

**Keywords:** obstructive sleep apnoea, implantable loop recorder, arrhythmia, atrial fibrillation, continuous positive airways pressure

## Abstract

**Background:**

Obstructive sleep apnoea (OSA) is highly prevalent in Western populations, causing breathing cessation during sleep due to airway collapse. OSA is strongly associated with cardiovascular disease and arrhythmia, with several mechanisms likely to increase arrhythmia incidence. Continuous positive airway pressure (CPAP) is the mainstay of treatment, and whilst CPAP effectively treats OSA, specific arrhythmias and major adverse cardiovascular events may remain unchanged. Furthermore, arrhythmias are likely significantly underdiagnosed in this population. Meanwhile, implantable loop recorders (ILRs) are the gold-standard detection method for arrhythmias.

**Methods:**

This review aimed to systematically evaluate observational studies using ILRs to identify the incidence of arrhythmia in treated OSA patients. We searched the Medline/Excerpta Medica Database (EMBASE) databases, identifying observational studies involving any OSA patients with no history of arrhythmia and who had ILRs inserted. Two reviewers assessed the quality of the studies and potential bias using the Observational Study Quality Evaluation (OSQE) tool.

**Results:**

Three studies met the criteria with 77 participants; however, the study outcomes were incomparable and could not be pooled. CPAP significantly reduced bradyarrhythmia/pauses. There was a high incidence of atrial fibrillation (AF), up to 31%, although the sample size and overall characteristics were insufficient and could not be generalized. AF and other tachyarrhythmias were likely underdiagnosed in OSA patients. CPAP did reduce bradyarrhythmia/pauses but is potentially insufficient to reduce AF or other tachyarrhythmias. Only one ongoing study was found to evaluate the incidence of arrhythmias in OSA.

**Conclusions:**

We highlight the need for arrhythmia screening in OSA patients and for further studies to clarify the true incidence of arrhythmias in OSA patients. These additional studies may influence the guidelines for arrhythmia screening and identify mechanisms and therapeutic targets in OSA.

## 1. Introduction

Obstructive sleep apnoea (OSA) is highly prevalent condition that affects ~1 billion adults worldwide and affects 1.4 million people in the UK alone [1]. It is hugely under diagnosed, especially among certain racial and ethnic groups. It is caused by obstruction or collapse of the pharyngeal airway during sleep with resultant apnoea and hypopnea episodes occurring repetitively [1]. This can be indexed over one hour and forms the basis of the diagnostic criteria of OSA, the apnoea hypopnea index (AHI). OSA prevents recuperative sleep and results in daytime somnolence, impaired quality of life and increased cardiovascular morbidity and mortality. It is strongly associated with obesity, cardiovascular diseases (including stroke) and diabetes and therefore represents a significant health burden [2].

Cardiac arrhythmias such as atrial fibrillation (AF), bradycardia, ventricular arrhythmias and sudden cardiac death are co-prevalent and co-incident with OSA, as well as cardiovascular and metabolic diseases [3]. In the Sleep Heart Health Study, incidence of nocturnal arrhythmias, including AF and ventricular arrhythmias, were 2–4 times more likely [4] and cardiovascular disease increased with AHI [5]. Whilst continuous positive airways pressure (CPAP) has been shown to reduce sleep apnoea symptoms, AHI, incidence of nocturnal pauses and recurrence of atrial fibrillation in smaller studies, large randomised control trials [6] and meta-analyses [7, 8] have not shown any significant reduction in incident AF, metabolic outcomes or cardiovascular disease.

OSA is linked to arrhythmia in both directions. OSA causes repeated hypoxia, hypercapnia and raised intrathoracic pressure resulting in oxidative stress, inappropriate activation of the autonomic nervous system and inflammation [9]. These factors have been shown to increase the likelihood of arrhythmia [10].

Most detections of arrhythmia occur after symptoms are described by patients and use snapshot monitoring for detection of arrhythmias including 10-second 12-lead electrocardiograms (ECGs) and 24-hour Holter monitoring. Others are found surreptitiously, for example, during a pre-operative assessment or overnight polysomnography on 12-lead ECG or single lead monitoring. However, the level of clinically significant arrhythmia in OSA patients is likely significantly underestimated [1] with few studies using longer-term arrhythmia monitoring in order to detect these paroxysms. Studies with implanted cardiac monitors such as pacemakers (with atrial leads) or implantable loop recorders (ILRs) demonstrate much higher pick-up rates of clinically significant but silent arrhythmias [11], particularly when looking for AF in cryptogenic stroke [12]. They offer a highly sensitive continuous long term heart rhythm monitoring which are far superior to traditional heart rhythm monitoring such as 24-hr Holter tapes.

Despite the latest evidence that OSA and AF in a treated OSA population are underdiagnosed and that there is a strong interplay between OSA and AF [13], National Institute for Health and Care Excellence (NICE) guidelines in the National Health Service (NHS) do not encourage screening for arrhythmias in a sleep apnoea cohort [14] and European Society of Cardiology (ESC) guidelines for AF only opportunistic screening for AF in OSA cohorts [15].

This review assesses the evidence base for arrhythmia detection using the current gold standard of an ILR for detection of arrhythmias in an OSA population. Our objective was to identify the incidence of arrhythmias in OSA patients as measured by implanted long-term cardiac monitors. Our study aims to systematically review observational studies to evaluate the incidence of arrhythmias detected by ILR in OSA patients and explore the potential effects of CPAP therapy on arrhythmias.

## 2. Methods

We performed a systematic search of Medline and Excerpta Medica Database (EMBASE) from 1974 to 2022 to identify observational studies which evaluated arrhythmia using ILRs in moderate-severe OSA patients. Most studies on arrhythmias in OSA patients rely on short-term Holter monitoring, potentially underestimating the prevalence of asymptomatic atrial fibrillation and other arrhythmias, while studies using ILR remain scarce.

### 2.1 Population, Intervention, Comparator and Outcomes (PICO)

A PICO strategy was developed to identify search terms for observational studies. The PICO terms were obstructive sleep apnoea patients, implantable loop recorder, and incidence of arrhythmia respectively. The variances of terms for OSA and ILR were accounted for in the search as described below. All known models of ILR were included in the search terms.

### 2.2 Search Strategy

The primary reviewer performed a search of Medline and EMBASE via Ovid. The searches were restricted to trials published in scientific peer reviewed journals involving human participants from 1974 to 30th March 2022. The search terms were “obstructive sleep apnoea” and “implantable loop recorder” combined with the ‘OR’ function with “obstructive sleep apnea”, “sleep apnoea”, “sleep apnea”, and “implantable cardiac monitor”, “insertable cardiac monitor”, “insertable loop recorder”, “subcutaneous cardiac rhythm monitor”, “reveal LINQ”, “biomonitor”, “confirm rx”, “lux dx” respectively. Our own knowledge of relevant papers and references from prior searches (including PubMed, EMBASE, ClinicalTrials.gov (https://clinicaltrials.gov/), Scholar, Cochrane Library, and Web of Science) were included.

### 2.3 Study Selection Criteria

Abstracts were screened independently by two reviewers with randomised control trials, case-control, and cohort studies, case-series and case studies included. Non-human studies, non-English language studies, reviews, letters, abstract only and conference papers were excluded from this study. Abstracts for which no full-text article were available to the reviewers were excluded from the study.

**Fig. 1.**
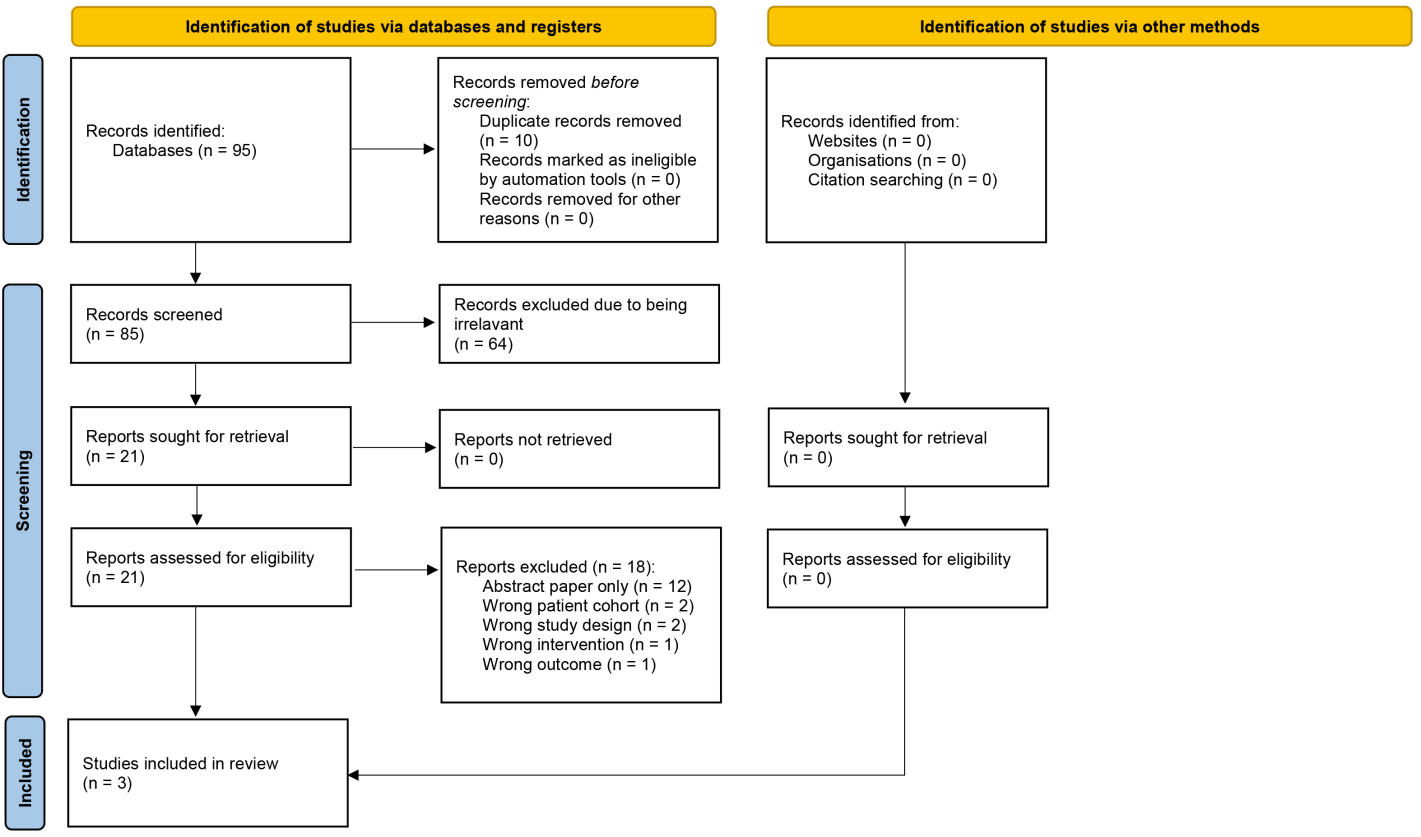
**Preferred Reporting Items for Systematic Reviews and Meta-Analyses (PRISMA) flow chart of study selection**.

Full-texts articles were reviewed to identify studies meeting the inclusion criteria of patients with moderate to severe obstructive sleep apnoea (AHI ≥15) whilst excluding articles which had patients with a past medical history of arrhythmia, and patients who already had an ILR or other types of extended cardiac monitoring. Studies were grouped by tachyarrhythmia and bradyarrhythmia. Conflicts at each stage were resolved in meetings of the reviewers. The Preferred Reporting Items for Systematic Reviews and Meta-Analyses (PRISMA) flow chart details and demonstrates this process (Fig. 1).

### 2.4 Risk of Bias and Quality Assessment

The Observational Study Quality Evaluation (OSQE) [16] is a risk of bias assessment tool specifically designed for systematic reviews of observational studies. The OSQE tool (See **Supplementary materials**) is specifically designed for observational studies, enabling comprehensive assessment of study representativeness, variable validity, loss of follow-up, and analysis, and risk of bias (reporting or conflict of interest), making it suitable for this systematic review. A star is attributed to the study based on questions within each of the above domains with a maximum score of 15 for this review.

Other well-known risks of bias assessments (e.g., ROBINS-I) were based on reviews conducted on randomised control trials and whilst many points were analogous, others did not apply or were confusing to use in evaluating observational studies. The OSQE tool provided in the supplementary material was applied by both reviewers to each study. Consensus was reached between the reviewers with overall grades demonstrated in Table 1 (Ref. [17, 18, 19]).

**Table 1.** **Risk of Bias and Quality Analysis by OSQE**.

Study	Representativeness	Independent Variable	Dependent Variable	Loss to follow-up	Miscellaneous	Overall
	/2	/2	/5	/2	/4	/15
Simantirakis *et al*. 2004 [17]	2	2	3	2	1	10
Healey *et al*. 2017 [18]	1	0	4	1	1	7
Yeung *et al*. 2018 [19]	2	2	4	2	3	13

OSQE, Observational Study Quality Evaluation. Max Score, Miscellaneous includes analysis, reporting or conflict of interest bias.

### 2.5 Data Extraction and Analysis

Each reviewer independently extracted data points including author details, patient demographics, medical history, and arrhythmia incidence. Any conflicts were resolved by the primary author after reviewing the original papers again. Full details of data extracted are included in Tables 2,3 (Ref. [17, 18, 19]). Each paper identified in the study was reviewed for methodological quality and comparisons made between the population demographics, and outcomes, if appropriate.

**Table 2.** **Description of included studies**.

Study	Country	Study Type	Inclusion, n	Exclusions	Primary Outcomes
Simantirakis *et al*. 2004 [17]	Greece	Cohort	Moderate to severe OSA patients (AHI 15–30, AHI ≥30), 23	HTN, IHD, arrhythmia, diabetes, COPD, cardioactive medications	Brady and tachy-arrhythmias
Healey *et al*. 2017 [18]	Canada, Netherlands	Cohort	Patients older than 65 with risk factors for but no AF - subgroup of OSA patients, 29	AF, Atrial Flutter, PPM, CRT or CRT (planned or otherwise), Oral Anticoagulation	Sub-clinical AF 5 mins
Yeung *et al*. 2018 [19]	Canada	Cohort	Severe OSA patients (AHI ≥30), 25	AF, heart failure, other cardiac device	AF ≥10 s

OSA, obstructive sleep apnoea; AHI, apnoea-hypopnoea index; HTN, hypertension; IHD, ischaemic heart disease; COPD, chronic obstructive pulmonary disease; AF, atrial fibrillation; PPM, permanent pacemaker; CRT, cardiac resynchronisation therapy.

**Table 3.** **Sample characteristics for included studies**.

Study	Mean F/U	Age ± SD	Sex	Mean AHI ± SD	CPAP Therapy	Relevant reported characteristics (Mean or %)		ILR type
Simantirakis *et al*. 2004 [17]	16	50 ± 8	9F, 14M	61 ± 23	Yes	BMI	33–37 ± 5	Unknown
						LVEF	61–63 ± 3.7	
Healey *et al*. 2017 [18]	16.3 ± 3.8	Unclear	Unclear	Unknown	Unknown	Not discernable from presented data		St. Jude Medical CONFIRM-AF
Yeung *et al*. 2018 [19]	27	57 ± 10	12F, 13M	55 ± 18	Yes	BMI	35 ± 6	Medtronic REVEAL-XT
						Hypertension	56%	
						Diabetes	12%	
						Stroke	0%	
						CAD	24%	
						COPD	8%	

F/U, follow-up; AHI, apnoea-hypopnoea index; CPAP, continuous positive airways pressure; ILR, implantable loop recorder; F, female; M, male; BMI, body mass index; LVEF, left ventricular ejection fraction; CAD, coronary artery disease; COPD, chronic obstructive pulmonary disease.

Arrhythmia outcomes were displayed as categorical frequencies and percentages. Grouped odds ratios were displayed for the effect of CPAP treatment on reducing arrhythmia as appropriate. Missing data points were highlighted in the relevant tables and noted if that cohort were pooled in analysis. Due to the high heterogeneity among included studies, we did not perform quantitative meta-analysis but instead adopted a qualitative synthesis approach to describe the main results.

### 2.6 Ongoing and Unpublished Studies

Further to the protocol, a search of ‘not yet recruiting, recruiting, enrolling by invitation, or completed’ studies on clinicaltrials.gov and World Health Organization International Clinical Trials Registry Platform (WHO ICTRP) was performed with search terms ‘sleep apnoea’ and ‘arrhythmias, cardiac’ in March 2023. The summaries of each study were reviewed by the main author.

Selection for analysis was made based on inclusion and exclusion criteria outlined in ‘Study Selection’. Additionally, studies already found in the literature search or were withdrawn from ClinicalTrials.gov were excluded.

## 3. Results

### 3.1 Search Description

The search protocol yielded 95 records of which 10 were automatically identified as duplicates. 85 abstract and titles were screened with 64 excluded as determined by our exclusion criteria. Full-text articles were available for all 21 remaining articles. The full texts were reviewed independently with the 18 exclusions detailed in Fig. 1. Three studies [17, 18, 19] were taken through for risk of bias assessment (OSQE Score — Table 1) and data extraction. Inter-rater reliability at each stage is recorded in the supplementary material.

### 3.2 Characteristics of Included Studies

Three cohort studies were included in this review with 77 OSA patients of varying severity and from 2004 to 2018. The three countries were Greece [17], Canada [18, 19] and The Netherlands [18]. The factors controlled for included age, comorbidities, cardiac devices or planned cardiac device are detailed in Table 2. The patient characteristics are detailed in Table 3. Of note, Healey *et al*. [18] described a subgroup of OSA patients of which subgroup characteristics were not available and mean age of the overall population was 74 compared to 50 and 57 for Simantirakis *et al*. [17] and Yeung *et al*. [19] respectively.

Simantirakis *et al*. [17] did not identify the type of loop recorder used; both Healey *et al*. [18] and Yeung *et al*. [19] utilised the St. Jude Medical Confirm and Medtronic REVEAL XT ILR devices respectively. These devices were implanted with the sole purpose of identifying arrhythmias in a cohort who did not have any criteria for ILR implantation. Healey *et al*. [18] and Yeung *et al*. [19] had their ILRs set to maximise sensitivity for AF and therefore did not report any other arrhythmias. These parameters are also well defined and therefore detection of AF can be adequately determined. Only Yeung *et al*. [19] detailed the co-morbidities and characteristics of their cohort sufficiently for a comparison to be made.

### 3.3 Risk of Bias and Quality Analysis

The final OSQE score for each study is shown in Table 1. Question 15 was not used in this study as effect modifiers were not relevant. The maximum score is therefore 15. Representativeness was demonstrated by two studies [17, 19] with the optimum population without too many exclusions. Healey *et al*. [18] demonstrated a very wide range of patients with only a small proportion of patients (OSA specifically) relevant to this review. The independent variables were adequately judged and exposed only by Simantirakis *et al*. [17] and Yeung *et al*. [19]. Healey *et al*. [18] did not detail how the OSA subgroup was identified simply by a medical history and therefore the parameters of this subgroup could not be identified.

The identification of arrhythmias was the dependent variable in each study but differed in type between the studies making comparison or pooling of data impossible. None were blinded (as expected) but all had adequate follow-up and continuous assessment. Follow-up was sufficient in each study (loss of follow-up all below 10%), however it is unclear how Healey *et al*. [18] dealt with missing data. Conflict of interest was potentially present for Healey *et al*. [18] and there were no details in Simantirakis *et al*. [17]. Confounders were addressed only by Yeung *et al*. [19]. All studies reported results as per their protocol. Simantirakis *et al*. [17] acknowledged an inadequate sample size. The others did not comment. Due to the non-comparable patient cohorts, high heterogeneity of devices used, heterogeneity of the outcomes and the low number of relevant studies, the data could not be pooled a meta-analysed. Instead, a qualitative analysis approach was taken to describe the results.

### 3.4 Primary Outcomes

Atrial fibrillation was detected in 20*–*31% of patients in two of the studies. In one study supraventricular tachycardia (SVT) occurred in 13% (Table 4, Ref. [17, 18, 19]). Simantirakis *et al*. [17] report over half of their patients (56%) experiencing bradycardias only prior to CPAP treatment. These bradycardic events were eliminated after 10 months of CPAP treatment (*p* = 0.028).

**Table 4.** **ILR Arrhythmia findings from included studies**.

Study	n	Bradycardia/Pauses	AF	Other
Simantirakis *et al*. 2004 [17]	23	9 (56%)*	0	2 SVT (13%)*
Healey *et al*. 2017 [18]	29	0^†^	9 (31%)	0^†^
Yeung *et al*. 2018 [19]	25	0^†^	5 (20%)	0^†^

*before CPAP treatment. † ILR was set for AF detection only. ILR, implantable loop recorder; n, number; AF, atrial fibrillation; SVT, supraventricular tachycardia; CPAP, continuous positive airways pressure.

CPAP treatment did not reduce SVT occurrence statistically. Yeung *et al*. [19] also found no reduction in AF with CPAP treatment. All cohorts of the included papers were small and not generalisable to other populations and therefore we cannot draw any certainty from their results without further studies adding to this data.

### 3.5 Search Description of Ongoing and Unpublished Studies

There were 73 results from the initial clinicaltrials.gov search and 7 from WHO ICTRP. All 7 trials from WHO ICTRP were from extracted from clinicaltrials.gov and therefore excluded. Two studies were duplicates and another had already been included in the main analysis; 44 studies were not relevant. Twenty-six studies had at least one sleep apnoea group who were being monitored for 48 or more hours (using various devices). However, these studies were primarily looking at recurrence in an AF population and a few were looking at occurrence in specific populations such as bradycardic patients, those patients undergoing AF ablation or patients with heart failure but not specifically sleep apnoea. Two studies met the study selection criteria of which one is completed and other currently recruiting.

### 3.6 Characteristics of Included Ongoing and Unpublished Studies

The first relevant study, Arrhythmia Detection In Obstructive Sleep Apnoea (ADIOS) [20] started on 6th March 2017 and was completed on 3rd April 2019. The study was performed by the University of Miami, Florida, USA. They enrolled 86 patients (with 1 drop-out) between the ages of 40 yrs and 85 yrs with newly diagnosed OSA without AF. A 2-week cardiac event monitor (Lifestar Act III) was applied. The mean age was 59.3 years with a 46:40 female-to-male ratio. Two patients (2.4%) were diagnosed with AF; 22 (25.9%) had frequent premature ventricular contractions and 12 (14.1%) had frequent premature atrial contractions. Other outcomes such as AF greater than 6 minutes and longest duration of AF were collected but were not published on clinicaltrials.gov. There were no adverse events. The data was not published in a peer-reviewed journal to the best of our knowledge.

The second relevant study is our own study, the Obstructive Sleep Apnoea and Cardiac Arrhythmias (OSCA) trial [21] that started recruiting in October 2019 and is a randomised nested controlled trial that is currently ongoing. It is being performed at University Hospitals Coventry and Warwickshire NHS Trust, UK with an expected end date in 2025. The enrolment target is 200 with patients having at least moderate OSA and requiring CPAP therapy. No documented arrhythmias are allowed at the start and patients are between the ages of 18 yrs and 75 yrs. One arm received standard care alone and the intervention arm received an ILR (Medtronic REVEAL LINQ). The primary outcome is the incidence of arrhythmias with secondary outcomes looking at cardiovascular mortality and morbidity, AF burden, cardiac autonomic function, cardiovascular biomarkers and quality of life both before and after CPAP therapy [22].

## 4. Discussion

Despite evidence that CPAP does not reduce tachyarrhythmia incidence and that traditional investigations for arrhythmia (short-term intermittent Holter monitoring) will not identify the full spectrum of arrhythmia incidence, there are very few studies that have investigated the incidence of arrhythmia in an OSA population using ILRs which are much more sensitive. This systematic review demonstrated a total of only 77 patients enrolled in observational studies using ILRs which forms only a tiny proportion of OSA patients worldwide, and a single unpublished study using shorter term monitoring with findings similar to the current literature.

Whilst these numbers are small and the tachyarrhythmia findings not statistically significant, they provide tantalizing evidence that AF is significantly under diagnosed in severe OSA patients (up to 31% compared to 5% in a large study with traditional AF detection [4] even though the diagnostic criteria differed. This could explain, in part, why CPAP did not significantly reduce major adverse cardiovascular events (MACE) in the largest CPAP trial to date [6] and is supported by findings from recent meta-analyses. Reduced CPAP compliance in these trials could also explain no significant reduction [23]. Yeung *et al*. [19] measured CPAP adherence, splitting adherence into total, partial and non-adherence but did not define this, however, they did not find that CPAP adherence affected the risk of AF. Larger studies are needed to determine the need and the method for detecting AF and other tachyarrhythmias which could change current screening guidelines in OSA and whether CPAP has a significant effect. The one ongoing study described above (OSCA trial) [22] may provide more significant evidence.

Simantirakis *et al*. [17] did provide strong evidence that CPAP can eliminate bradycardias and pauses which is consistent with large studies [23] in this area that did not use ILRs, which is also reflected in currently known literature. However, there is a lack of understanding of the underlying mechanisms that link OSA and tachyarrhythmias [24]. Wider ranging studies which look at the metabolic state, cardiac autonomic function and incidence of all arrhythmias, could untangle this and lead to a stronger understanding and better targeted screening for OSA patients. Additionally, the mechanisms underlying these ongoing arrhythmias in OSA, despite CPAP treatment, needs to be further elucidated. There are several upcoming studies that will hopefully be able to answer these questions.

One of these studies is currently being undertaken by our group, in newly diagnosed moderate to severe OSA patients just prior to commencing CPAP therapy. This prospective nested controlled cohort study randomised these patients to ILRs and Holter monitoring. Participants body measurements, cardiovascular symptoms, cardiovascular biomarkers, cardiac autonomic function and crucially CPAP usage were recruited. The study aims to identify the true incidence of arrhythmias in this OSA population and look at the relationship of arrhythmia burden, CPAP usage, cardiac autonomic function and oxidative stress in order to identify particular subgroups or markers which significantly increase the risk of cardiac arrhythmias [22]. The study is expected to report in late 2025.

### Limitations

A protocol as per PRISMA guidance was not submitted due to the anticipated small scope and number of studies and therefore not registered with PROSPERO. This may introduce bias into the study selection process without protocol defined exclusion criteria. However, the exclusion criteria were pre-agreed by the reviewers and applied in the study selection software. Due to the above reasons this review was not registered. There were only three studies included so no significant comparisons could be made given the relatively small numbers enrolled, therefore each study was individually appraised. Some studies appear to have been completed but did not have any published results, thereby potentially biasing positive findings.

The studies included did not all report relevant baseline characteristics of their populations and heterogeneity, which may be related to differences in ILR parameter settings and baseline characteristics of patients. Combined with the small number of studies and relatively small patient numbers in each study, no meta-analysis was possible. All of these studies were observational as by the nature of this study, therefore identifying OSA or poor CPAP compliance as a cause of arrhythmia was not possible There was no relevant evidence on incident ventricular arrhythmias. It was not clear why Simantirakis *et al*. [17] did not identify the model of ILR used in their study. Although this could inhibit the reproducibility of the study, they did state the parameters of detection for the device.

## 5. Conclusions

There are not enough published studies currently to clearly identify the true incidence of arrhythmias in OSA patients treated with CPAP, although there is a signal that CPAP does not ameliorate the tachyarrhythmia risk, particularly AF. However, the risk of asymptomatic AF appears to be significant and should be screened for as part of a comprehensive OSA assessment. This approach may influence and improve outcomes for OSA sufferers. We recommend future studies looking into arrhythmias in OSA should include both established and novel diagnostic investigations such as extended cardiac monitoring (both internal and external) to explore underlying mechanistic links between OSA and arrhythmias. Studies should also include assessment of cardiac autonomic function (heart rate varibility and heart rate turbulence) and impact of effective CPAP therapy. The negative effects of oxidative stress on cardiovascular outcomes also need to be studied though changes in circulating vascular biomarkers such as interleukin-6, tumour necrosis factor alpha, high sensitivity C reactive protein (CRP) microRNAs and other emerging stress biomarkers. The mechanisms underlying these arrhythmias are not yet well understood. Trials are currently underway with results eagerly anticipated in due course. Our review highlights the importance of routine arrhythmia screening in OSA patients, particularly the high incidence of asymptomatic atrial fibrillation, which may influence treatment decisions and improve outcomes.

## Availability of Data and Materials

The data underlying this article will be shared at request to the corresponding author.

## Supplementary Material

Supplementary material associated with this article can be found, in the online version, at https://doi.org/10.31083/RCM31308.
