# Supplementary material for: Incident Arrhythmias Detected Using Implantable Loop Recorders in Obstructive Sleep Apnoea
Source: Rev Cardiovasc Med. 2025 Jul 11;26(7):31308. doi: 10.31083/RCM31308 (PMC12326399; doi:10.31083/RCM31308)
Supplement: Supplementary file 1 [file 2153-8174-26-7-31308-s1.zip › Supplementary materials.docx]

# SUPPLEMENTARY MATERIALS

## Search Strategy

Embase <1974 to 2022 March 30>

Ovid MEDLINE(R) ALL <1946 to March 30, 2022>

1 Obstructive Sleep Apnoea.mp. [mp=ti, ab, hw, tn, ot, dm, mf, dv, kf, fx, dq, nm, ox, px, rx, ui, sy] 14314

2 Implantable Cardiac Monitor.mp. [mp=ti, ab, hw, tn, ot, dm, mf, dv, kf, fx, dq, nm, ox, px, rx, ui, sy] 12993

3 Implantable Loop Recorder.mp. [mp=ti, ab, hw, tn, ot, dm, mf, dv, kf, fx, dq, nm, ox, px, rx, ui, sy] 1793

4 Reveal LINQ.mp. [mp=ti, ab, hw, tn, ot, dm, mf, dv, kf, fx, dq, nm, ox, px, rx, ui, sy] 330

5 Insertable Cardiac Monitor.mp. [mp=ti, ab, hw, tn, ot, dm, mf, dv, kf, fx, dq, nm, ox, px, rx, ui, sy] 416

6 Insertable Loop Recorder.mp. [mp=ti, ab, hw, tn, ot, dm, mf, dv, kf, fx, dq, nm, ox, px, rx, ui, sy] 85

7 Extended cardiac monitoring.mp. [mp=ti, ab, hw, tn, ot, dm, mf, dv, kf, fx, dq, nm, ox, px, rx, ui, sy] 55

8 Sleep Apnoea.mp. [mp=ti, ab, hw, tn, ot, dm, mf, dv, kf, fx, dq, nm, ox, px, rx, ui, sy] 18809

9 Sleep Apnea.mp. [mp=ti, ab, hw, tn, ot, dm, mf, dv, kf, fx, dq, nm, ox, px, rx, ui, sy] 123814

10 Obstructive Sleep Apnea.mp. [mp=ti, ab, hw, tn, ot, dm, mf, dv, kf, fx, dq, nm, ox, px, rx, ui, sy] 72856

11 1 or 8 or 9 or 10 131779

12 biomonitor.mp. [mp=ti, ab, hw, tn, ot, dm, mf, dv, kf, fx, dq, nm, ox, px, rx, ui, sy] 2059

13 confirm rx.mp. [mp=ti, ab, hw, tn, ot, dm, mf, dv, kf, fx, dq, nm, ox, px, rx, ui, sy] 69

14 lux dx.mp. [mp=ti, ab, hw, tn, ot, dm, mf, dv, kf, fx, dq, nm, ox, px, rx, ui, sy] 3

15 subcutaneous cardiac rhythm monitor.mp. [mp=ti, ab, hw, tn, ot, dm, mf, dv, kf, fx, dq, nm, ox, px, rx, ui, sy] 4

16 2 or 3 or 4 or 5 or 6 or 7 or 12 or 13 or 14 or 15 16425

17 11 and 16 95

## OSQE Quality Evaluation Tool (Cohort Studies)

https://www.frontiersin.org/articles/10.3389/frma.2021.675071/full
